# Temporal Trends and Early Outcomes of Transcatheter versus Surgical Mitral Valve Repair in Atrial Fibrillation Patients

**DOI:** 10.1155/2023/4332684

**Published:** 2023-10-12

**Authors:** Chi Zhou, Kai Tan, Weili Liu, Shaohua Li, Zongyi Xia, Yanxu Song, Zhexun Lian

**Affiliations:** ^1^Department of Cardiology, The Affiliated Hospital of Qingdao University, Qingdao, China; ^2^Interventional Operation Room, The Affiliated Hospital of Qingdao University, Qingdao, China

## Abstract

**Objectives:**

To study trends of utilization, in-hospital outcomes, and short outcomes in patients undergoing transcatheter mitral valve repair (TMVR) vs. surgical mitral valve repair (SMVR) in atrial fibrillation (AF).

**Background:**

TMVR is a treatment option in inoperable or high-risk patients with mitral regurgitation (MR). AF is a common comorbidity of MR. Data comparing between TMVR and SMVR in MR patients with AF is lacking.

**Methods:**

The National Readmission Database from 2016 to 2019 was utilized to identify hospitalizations undergoing TMVR or SMVR with AF. Outcomes of interest included mortality, postoperative complications, length of stay, and 30-day readmission rate.

**Results:**

A total of 9,195 patients underwent TMVR and 16,972 patients underwent SMVR with AF; the number of AF undergoing TMVR was increasing from 1,342 in 2016 to 4,215 in 2019 and SMVR. The incidence of in-hospital mortality decreased from 2.6% in 2016 to 1.8% in 2019. We identified length of stay>5 days, dyslipidemia, cerebrovascular disease, heart failure with reduced ejection fraction, and urgent/emergent admissions as independent risk factors for in-hospital mortality. After matching, we included 4,680 patients in each group; the in-hospital death, transfusion, acute kidney injury, sepsis, stroke, and mechanical ventilation were lower in TMVR compared with SMVR. TMVR was associated with a similar rate of all-cause readmission at 30 days compared with SMVR.

**Conclusion:**

Patients with AF receiving TMVR have been increasing along with progressive improvement in in-hospital death and length of stay. Compared to SMVR, AF patients receiving TMVR had a lower rate of in-hospital death and postoperative complications.

## 1. Introduction

Mitral regurgitation (MR) is a common heart valve disease that affects approximately 10% of people over the age of 75 in the general population [1]. Severe MR can lead to left ventricular failure, pulmonary hypertension, atrial fibrillation, and even death, and in patients with a New York Heart Association (NYHA) class III or IV, the mortality rate is 34% yearly [2]. Surgical mitral valve repair (SMVR) used to be the only treatment option for patients with MR that was poorly controlled by medications [3, 4]. In 2003, the first MitraClip procedure was performed and received European CE certification in 2008. Based on the results of the EVEREST II and COAPT clinical trials, transcatheter mitral valve repair (TMVR) for primary and secondary MR has been updated to class 2a in the latest guidelines.

Atrial fibrillation (AF) is often associated with valvular heart disease (VHD), and up to 60% of the patients with AF have some form of valvular abnormality [5]. Previous research has revealed poor outcomes in AF patients treated with SMVR [6]. Patients with AF who undergo TMVR have higher risks of postoperative bleeding and stroke as well as a higher risk of mortality during follow-up. As TMVR is being increasingly performed, its safety in specific patient populations needs further clarification. However, there is very little published research specifically comparing the outcomes of TMVR and SMVR in AF patients [7]. This study compared utilization trends and outcomes of TMVR and SMVR using nationally representative real-world population data.

## 2. Methods

### 2.1. Data Source

The National Readmission Database (NRD) from January 2016 to December 2019 was used for this analysis. The NRD is a part of administrative databases developed by federal support of Healthcare Research and Quality (AHRQ) agency. The NRD has over 16 million admissions, which represents a 57% stratified sample of all discharges from community hospitals in the United States excluding rehabilitation and long-term acute care hospitals [8]. As an administrative database, the NRD used the International Classification of Diseases, 10th Revision (ICD-10) code, to identify patient diagnosis and operation. This article was approved by the Ethics Committee of the Affiliated Hospital of Qingdao University (ethics number: QYFY WZLL 27354).

### 2.2. Study Design and Data Selection

We searched the 2016–2019 NRD for patients with AF who underwent SMVR or TMVR using the ICD-10 (TMVR: 02UG3JZ; SMVR: 02QG0ZE or 02QG0ZZ). Patients with less than 18 -year-old and missing data were excluded. Meanwhile, we also excluded patients with CABG to reduce the likelihood of selection in favor of SMVR. To calculate the estimated cost of hospitalization, the NRD data were merged with cost-to-charge ratios available from the Healthcare Cost and Utilization Project. We estimated the cost of each inpatient stay by multiplying the total hospital charge with cost-to-charge ratios. Cost was also adjusted for inflation (December 2021).

### 2.3. Outcomes

Our study's primary outcome was in-hospital outcomes including the in-hospital death, length of stay, cost, transfusion, acute kidney injury, sepsis, mechanical ventilation, and stroke. The secondary outcomes were the 30-day outcomes including all-cause readmission, cardiovascular (CV) readmission, and heart failure readmission. CV readmission was defined as hospitalization due to myocardial infarction, heart failure, or arrhythmia. The primary diagnosis in the first readmission after TMVR or SMVR was a reason for readmission. In the case of multiple postoperative readmissions, only the first hospitalization after admission was counted. All ICD-10 codes used for cohort screening, baseline characteristics, and outcomes are presented in Supplementary Table 1.

## 3. Statistical Analysis

Normally distributed measures are expressed as the mean ± variance using Student's *t* test; nonnormally distributed measures are expressed as the median (interquartile range (IQR)) using the Wilcoxon rank-sum test. Categorical variables are expressed as percentages, and comparisons between the groups were made using the *X*^2^ test or the Fisher's exact test. A nearest neighbor 1 : 1 variable ratio, parallel, balanced, propensity-matching model was made using a caliper width of 0.2. The Cochran–Armitage trend test was used for categorical variables and linear regression for continuous ones' trend analysis. We entered variables with *P* < 0.05 in the univariate analysis into a multivariate regression model and selected the entry method to identify independent predictors of in-hospital death and 30-day readmission and compute the odds ratio (OR) and 95% confidence interval (CI).

We chose two sides' *P* value of <0.05 as statistically significant. We used univariate Cox proportional hazards regression for the 30-day outcomes to calculate the hazard ratio (HR) and 95% CI. Kaplan‒Meier curves for 30-day all-cause readmissions were constructed. Statistical analyses and propensity-matched analysis were performed using R version 3.5.

## 4. Result

### 4.1. Population Characteristics and Outcomes

**Figure 1:**
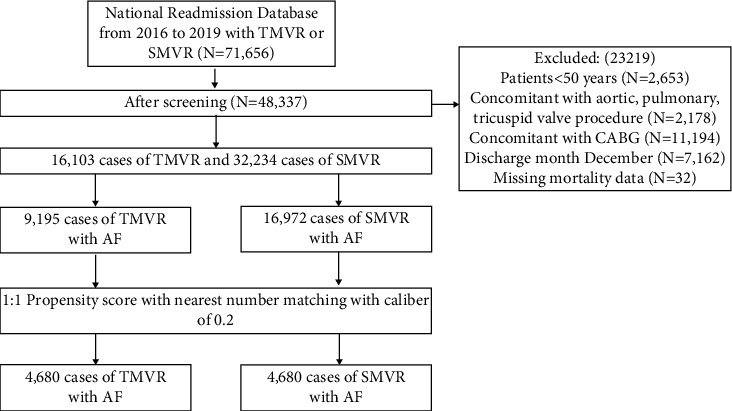
Diagram of patient screening.

There were 26,037 patients of TMVR and 45,619 patients of SMVR performed in the NRD between 2016 and 2019. After screening, we finally included 9,195 patients of TMVR and 16,972 patients of SMVR.  Figure 1 illustrates the patient selection process. Before matching, patients in the TMVR group were older (mean age of 79.3 years vs. 66.6 years; *P* < 0.001) and had more women (44.7 vs. 41.2%; *P* < 0.001) compared with those in the SMVR group. The TMVR group associated more comorbidities including the prior PCI, prior CABG, smoke, myocardial infarction, congestive heart failure, chronic pulmonary disease, diabetes, renal disease, cerebrovascular disease, peripheral vascular disease, and metastatic solid tumor compared with the SMVR group. Conversely, more obesity was associated in the SMVR group. The detailed differences between TMVR and SMVR are summarized in Table 1.

### 4.2. Temporal Trends

Over the study period, the number of AF undergoing TMVR was increasing from 1,342 in 2016 to 4,215 in 2019 (*P* = 0.049 for trend) and with SMVR remained unchanged from 4,323 in 2016 to 4,084 in 2019. In the TMVR group, we reported the in-hospital mortality in the range of 2% (2.6 vs. 1.8%; *P* = 0.049 for trend), while in SAVR, the mortality rate also declined (2.2 vs. 1.7%; *P* = 0.037 for trend). In terms of postoperative complications in the SMVR group, transfusion, sepsis, acute kidney injury, mechanical ventilation, and stroke all remained unchanged. However, in the TMVR group, the sepsis rate declined (2.7 vs. 1.0%; *P* = 0.037 for trend) and transfusion, acute kidney injury, mechanical ventilation, and stroke all remained unchanged.

In the 30-day readmission rate, the all-cause and CV reason remained stable, but there was a trend towards a decreased admissions for heart failure (2.7 vs. 1.0%; *P* = 0.037 for trend) in the TMVR group. However, in the SMVR group, the all-cause, CV, and heart failure reason rate all declined during 2016–2019. The TMVR and SMVR groups' temporal trends between 2016 and 2019 patients are summarized in Tables 2 and 3.

### 4.3. Multivariate Predictors of In-Hospital Death and 30-Day Readmission

In the TMVR group, among the univariate logistic regression analyses of risk factors for in-hospital mortality, we determined that length of stay (LOS) >5 days, dyslipidemia, cerebrovascular disease, heart failure with reduced ejection fraction, urgent/emergent admission, heart failure with preserved ejection fraction, renal disease, smoke, Charlson comorbidity index, paraplegia, and myocardial infarction had statistically significant differences. After adjusting for these confounding factors, we identified LOS >5 days, dyslipidemia, cerebrovascular disease, heart failure with reduced ejection fraction, and urgent/emergent admission as risk factors for in-hospital mortality.

In the TMVR group, among the univariate logistic regression analyses of risk factors for 30-day readmission, we determined that LOS >5 days, female, chronic pulmonary disease, dementia, heart failure with reduced ejection fraction, renal disease, urgent/emergent admission, heart failure with preserved ejection fraction, and Charlson comorbidity index had statistically significant differences. After adjusting for these confounding factors, we identified LOS >5 days, female, chronic pulmonary disease, dementia, heart failure with reduced ejection fraction, and renal disease as a risk factor for 30-day readmission. Tables 4 and 5 summarize the odds ratio and 95% CI both the univariate and multivariate logistic regression.

### 4.4. Outcomes in Matching Cohort

The variables used in the matching model are shown in the Supplementary Table 2. We have also shown the data distribution before and after propensity matching in the Supplementary Figure 1.

After matching, a total of 4,680 TMVR patients were matched with 4,680 SMVR patients with AF. All the baseline characters were equally balanced between the two groups (Table 1). TMVR was associated with a lower risk in the hospital death (2.3 vs. 3.3%; OR: 0.69; 95% CI: 0.53–0.88; *P* = 0.003). Meanwhile, TMVR had fewer surgical complications, including transfusion, acute kidney injury, sepsis, stroke, and mechanical ventilation. The TMVR group had shorter hospital stay and less cost. All the matching cohort outcomes are detailed in Tables 6 and 7.

### 4.5. 30-Day Outcomes

**Figure 2:**
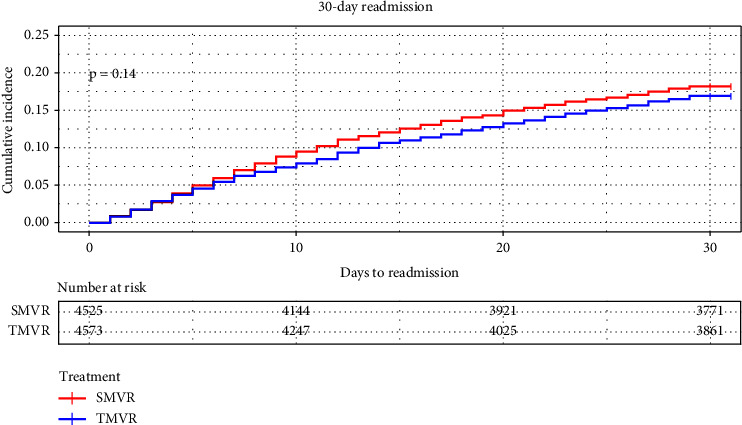
Kaplan–Meier graphs showing the 30-day all-cause readmission rates comparing SMVR with TMVR.

Patients undergoing TMVR had no difference in 30-day all-cause readmission (HR: 0.93; 95% CI: 0.84–1.03; *P* = 0.139), CV readmission (HR: 0.99; 95% CI: 0.75–1.02; *P* = 0.053), and heart failure readmission (HR: 0.88; 95% CI: 0.70–1.13; *P* = 0.89) compared with SMVR patients.  Figure 2 shows the 30-day all-cause readmission Kaplan–Meier curve. All the 30-day outcomes are detailed in Table 6.

### 4.6. Subgroup Analysis

According to the AF type, we divided the patients into paroxysmal AF and nonparoxysmal AF groups in the matching cohort. In the paroxysmal AF group, TMVR and SMVR had the similar risk in the hospital death and stroke. TMVR was associated with a lower rate in the transfusion, acute kidney injury, sepsis, and mechanical ventilation.

In the nonparoxysmal AF group, the TMVR and SMVR had the similar risk in sepsis and stroke. TMVR was associated with a lower rate in the hospital death, transfusion, acute kidney injury, and mechanical ventilation.. The paroxysmal AF detail outcomes are shown in Table 8, and the nonparoxysmal AF group outcomes are shown in Table 9.

### 4.7. MR Etiology

According to the different causes of mitral regurgitation, we classify them into rheumatic mitral valve, mitral valve prolapse, congenital heart disease, and other cause groups. In the rheumatic mitral valve group, TMVR and SMVR had the similar risk in the hospital death, sepsis, cardiogenic shock, and stroke. TMVR was associated with a lower rate in transfusion, acute kidney injury, and mechanical ventilation.

In the mitral prolapse group, TMVR and SMVR had the similar risk in the hospital death, mechanical ventilation, acute kidney injury, sepsis, and stroke. TMVR was associated with a lower rate in transfusion and cardiogenic shock.

In the congenital valvular heart disease group, TMVR and SMVR had the similar risk in the hospital death, mechanical ventilation, acute kidney injury, sepsis, transfusion, and cardiogenic shock.

In the other reason group, TMVR was associated with a lower rate in hospital death, mechanical ventilation, acute kidney injury, sepsis, transfusion, and cardiogenic shock. The different etiologies of mitral regurgitation outcomes are shown in Table 10.

## 5. Discussion

We report the following main findings in our contemporary real-world population study of TMVR and SMVR outcomes in patients with AF. (1) Patients with AF receiving TMVR have been increasing since 2016 and SMVR has remained stable. (2) The incidence of in-hospital mortality decreased from 2.6% in 2016 to 1.8% in 2019, and 30-day all-cause readmission remains stable in the TMVR group. (3) In AF receiving TMVR, LOS>5 days, dyslipidemia, cerebrovascular disease, heart failure with reduced ejection fraction, and urgent/emergent admission were associated with in-hospital mortality. LOS >5 days, female, chronic pulmonary disease, dementia, heart failure with reduced ejection fraction, and renal disease were associated with 30-day readmission. (4) In the matching cohort, compared to SMVR, AF patients receiving TMVR had a lower rate of in-hospital death, transfusion, acute kidney injury, sepsis, stroke, and mechanical ventilation. (4) In the subgroup analysis, in the paroxysmal AF group, TMVR and SMVR had the similar risk in hospital death, but in the nonparoxysmal AF group, TMVR was associated with a lower rate in hospital death. (5) In the 30-day outcomes, TMVR and SMVR had a similar result in all-cause readmission, heart failure readmission, and CV readmission.

TMVR is being increasingly performed because of its expanding real-world application and growing popularity. MR and AF are closely related. MR can lead to atrial enlargement, possibly resulting in an increased incidence of AF [9]. In a retrospective study in which echocardiographic characteristics were collected, researchers observed that the annual incidence of new-onset AF was approximately 5% in primary MR patients [10]. In previous trials and registry studies in which NRD was consulted, approximately 32 to 68% of AF patients underwent TMVR [11–14]. Using NRD, we identified in the real world about 57.1% of patients with MR who underwent mitral valve repair coexisting with AF. We identified independent risk factors for in-hospital mortality in patients with atrial fibrillation receiving TMVR, including the importance of urgent/emergent admissions and prior cardiovascular events. AF is an important risk factor for ischemic stroke, and these patients tend to have higher rates of embolism and bleeding. These patients tend to have a higher rate of perioperative bleeding. So, enhanced management of anticoagulants in patients with AF helps reduce in-hospital mortality in those patients receiving TMVR.

Similar to previous studies, our TMVR cohort was older (mean age 79.4) and had a higher prevalence of pre-existing comorbidities. Before matching, the patients in the TMVR group were generally in poor condition and the in-hospital mortality was slightly higher comparing the SMVR group, but not statistically different. After matching, TMVR demonstrated a lower in-hospital death and multiple in-hospital outcomes, as shown in the current study, including blood transfusion, acute kidney injury, sepsis, mechanical ventilation, cardiogenic shock, and stroke. This result is probably related to the fact that transcatheter manipulation is less invasive to the body. Fewer in-hospital complications were also reflected in the lower length of stay and cost of hospitalization in the TMVR group.

During the study period, we reported that the in-hospital mortality rate was 2% in AF patients who underwent TMVR. The in-hospital mortality rate found in our study was similar to that found in Alkhouli et al.'s study, which reported an in-hospital mortality of 2.47% for patients who underwent TMVR and were identified using the NRD database from 2014 to 2018 [15]. Although the difference in the incidence of postoperative complications was not significant, the gradual decrease in incidence indicates a gradual improvement in the safety of TMVR. This improvement in the safety of TMVR is also reflected in a gradual decrease in the length of stay over the study period. By using the STS/ACC TVT Registry, we found that as TMVR became more frequently performed and as operators became more experienced in performing TMVR, the procedural success improved and the complication rate decreased [16, 17]. Early all-cause readmission correlates to a poor prognosis. AF patients who underwent TMVR had a high 30-day readmission rate.

Previous studies have shown that heart failure is the most common reason for readmission of TMVR patients [18]. We found that the 30-day readmission heart failure rate declined from 2016 to 2019 in AF patients who underwent TMVR. This decreased rate is associated with reduced mitral regurgitation and relief of heart failure symptoms in AF patients with TMVR. However, we also note that the heart failure rate did not change significantly from 2017 to 2019. Whether this is related to the loose extension of TMVR indications to patients with functional MR, especially atrial functional MR, still needs further investigation in the future.

Whether AF has adverse effects on patients receiving mitral valve repair remains unclear. Alexiou et al.'s and Kessler et al.'s studies concluded that AF has a major negative impact in the long-term follow-up in patients undergoing SMVR [5, 6]. However, other reports have shown that the outcomes of SMVR are similar in patients with AF and those without AF [19, 20]. For patients who underwent TMVR, AF was not an independent risk factor for in-hospital death [21, 22], but it was an independent risk factor for 30-day readmission [18]. Therefore, it is important to identify risk factors for readmission of AF patients to reduce readmission after TMVR. The longer length of stay may be associated with the patients' poorer general condition, more comorbidities, and a higher probability of in-hospital complications, which are also associated with an increased 30-day readmission rate [23]. Strategies such as increased collaboration between pulmonologists and nephrologists, multidisciplinary evaluation of TMVR patients, and early discharge may help to reduce the rate of readmission of AF patients.

According to the well-known EVEREST II trial subgroup analysis, the AF patients in the TMVR and SMVR groups had similar in-hospital death rates and 1-year survival outcomes [24]. Our results were consistent with those of previous studies, and no significant differences were observed between TMVR and SMVR in terms of 30-day readmission after matching. However, before matching, the SMVR group had a lower 30-day readmission rate. In fact, after matching, the SMVR patients we matched were older, frailer, and had more comorbidities. As a result, the prognosis of SMVR was poorer.

We evaluated the impact of different AF types on outcomes, and we found that the paroxysmal AF group had similar risk in hospital death compared to SMVR, but the nonparoxysmal AF group had a lower in-hospital mortality rate. Generally, the prognosis of patients with paroxysmal AF tends to be positively correlated with the presentation of the frequency of AF.

Patients with nonparoxysmal AF have worsening heart failure symptoms, frailty, and a higher burden of noncardiovascular disease. This is also reflected in a meta-analysis by Ganesan et al. including approximately 100,000 patients receiving SMVR, where all-cause mortality was higher in nonparoxysmal AF than in paroxysmal AF [25]. However, in our univariate logistic regression analyses, the paroxysmal AF is not a protective factor for in-hospital death in AF patients undergoing TMVR and this is consistent with Gangani et al.'s study [26]. Also, in our subgroup analysis, we found that in the nonparoxysmal AF patients, TMVR appears to be more beneficial for patients compared to SMVR. However, this result still needs to be further demonstrated in a randomized trial. In a subgroup analysis by etiology, we found no significant difference in in-hospital death between the two groups in the comparison of rheumatic mitral valve, mitral valve prolapse, and congenital heart disease. We found that TMVR was more favorable than SMVR in patients with mitral regurgitation due to other causes (including left atrium enlargement for various reasons and mitral valve degeneration). We think that this is due to part of the group of small sample size.

### 5.1. Limitations

Our study has the following limitations. NRD used for this analysis is an administrative database, and we used the ICD-10 code to identify the patients and outcomes, so it may suffer from coding errors. However, the procedure code was used to recognize our cohort, and we expect the errors in the coding to be negligible. Meanwhile, our study cannot calculate the Society of Thoracic Surgeons (STS) score. However, in our study, we found that the TMVR group had a high burden of comorbidity; it reflects the current guideline that TMVR patients are at intermediate or high surgical risk. Whether the STS score can be an indicator of patient prognosis factor is unclear. Meanwhile, the RCT study should be conducted to analyze whether there is a difference between TMVR and SMVR in intermediate or high surgical risk patients in the future.

Second, although we performed subgroup analyses based on different MR causes, including rheumatic heart disease, congenital valvular heart disease, and mitral valve prolapse, other causes including the search for secondary mitral regurgitation due to enlargement cannot be analyzed. Meanwhile, we also can get the echocardiography data (including the effective aortic valve orifice area, ejection fraction, pulmonary artery pressure, left ventricular diameter, left ventricular end-diastolic volume, and MR degree). These unmeasured factors may influence the results of the analysis as confounding factors.

Third, our study is a retrospective analysis with the inherent disadvantages of retrospective analysis. However, randomized grouping cannot be done as in randomized controlled studies although we used propensity score matching to reduce the difference between the two groups.

Fourth, the NRD database does not include emergency and out-of-hospital deaths. It can only count patients admitted to the hospital in the same state. However, we expect such readmissions to be uncommon in patients undergoing aortic valve replacement. Meanwhile, we used the ICD-10 code to identify outcomes, like others studies used [27, 28]. The possibility exists that some patients were coded incorrectly; however, the frequency of a given outcome being miscoded is similar in a particular group. The strength of this study is the large sample and the multicenter retrospective study.

## 6. Conclusion

We report real-world data on in-hospital outcomes of TMVR and SMVR in AF patient. The number of AF undergoing TMVR was increasing. We also found that the incidence of in-hospital mortality and sepsis decreased but the 30-day all-cause readmission remained stable. LOS >5 days, cerebrovascular disease, urgent/emergent admission, prior stroke, and smoke were considered as risk factors for in-hospital mortality; female, chronic pulmonary disease, and renal disease were considered as risk factors for 30-day readmission. After matching, TMVR had a low risk in hospital death, transfusion, acute kidney injury, sepsis, stroke, and mechanical ventilation. However, TMVR had a similar 30-day all-cause readmission incidence compared to SMVR in AF patients.

## Figures and Tables

**Table 1:** Baseline characteristics for SMVR versus TMVR in patients with AF in unmatched and matched cohorts.

	Unmatched cohort		*P* value	Matched cohort		*P* value
	SMVR in AF	TMVR in AF		TMVR in AF	SMVR in AF	
Number	16,972	9,195		4,680	4,680	
Age	66.66 ± 10.74	79.34 ± 8.82	<0.001	75.24 ± 7.38	75.71 ± 9.73	0.009
Female	41.2%	44.7%	<0.001	47.2%	49.0%	0.086
Paroxysmal AF	55.1%	53.7%	0.027	41.4%	37.4%	<0.001
Median income range^*∗*^			<0.001			0.095
0–25th percentile	18.0%	19.1%		19.8%	19.4%	
25−50th percentile	23.1%	23.9%		23.3%	25.5%	
50−75th percentile	27.0%	28.0%		27.2%	26.5%	
75−100th percentile	31.9%	29.0%		29.7%	28.6%	
Hospital size			<0.001			<0.001
Small	5.9%	2.8%		4.6%	3.2%	
Medium	22.0%	20.9%		19.6%	23.2%	
Large	72.1%	76.3%		75.8%	73.6%	
Pay			<0.001			0.192
Medicare	58.8%	90.2%		85.8%	86.3%	
Medicaid	5.4%	1.6%		2.2%	2.6%	
Others	35.8%	8.2%		12.0%	11.0%	
Urgent/emergent	21.5%	25.6%	<0.001	26.1%	25.8%	0.777
Prior PCI	4.8%	18.8%	<0.001	11.0%	10.8%	0.817
Prior CABG	2.7%	19.9%	<0.001	7.8%	8.7%	0.134
Prior PPM/ICD	6.8%	26.2%	<0.001	15.5%	15.9%	0.609
Smoke	29.5%	34.0%	<0.001	32.3%	32.3%	0.965
Dyslipidemia	48.0%	60.1%	<0.001	55.3%	55.9%	0.618
Anemia	3.5%	5.2%	<0.001	4.8%	4.5%	0.557
Obesity	15.6%	10.6%	<0.001	14.0%	13.7%	0.697
Alcohol use	0.2%	0.1%	0.081	0.2%	0.1%	0.605
Prior stroke	7.3%	12.9%	<0.001	10.9%	11.3%	0.553
Myocardial infarction	5.8%	16.7%	<0.001	11.2%	10.8%	0.552
Congestive heart failure	56.7%	89.0%	<0.001	82.7%	81.4%	0.118
Peripheral vascular disease	11.1%	24.4%	<0.001	18.4%	17.5%	0.27
Cerebrovascular disease	5.0%	5.6%	0.055	5.9%	5.9%	1
Dementia	0.7%	3.3%	<0.001	2.1%	2.0%	0.77
Chronic pulmonary disease	18.0%	27.4%	<0.001	25.8%	25.2%	0.507
Disease	0.3%	0.6%	<0.001	0.6%	0.5%	0.889
Peptic ulcer disease	2.5%	3.2%	0.001	3.0%	3.3%	0.443
Liver disease	2.2%	2.6%	0.102	2.8%	2.5%	0.521
Diabetes	12.1%	13.2%	0.008	14.4%	15.3%	0.211
Paraplegia	1.2%	0.5%	<0.001	0.8%	0.9%	0.647
Renal disease	16.2%	41.7%	<0.001	32.4%	31.1%	0.169
Metastatic solid tumor	0.2%	0.6%	<0.001	0.4%	0.4%	0.867
Charlson comorbidity index	4.68 ± 2.00	6.90 ± 1.97	<0.001	6.32 ± 1.86	6.26 ± 1.86	0.128

Normally distributed continuous variables expressed as the mean value ± standard deviation and categorical variables were expressed as percentages. AF: atrial fibrillation; SMVR: surgical mitral valve repair; TMVR, transcatheter mitral valve repair; PPM: permanent pacemaker implant; ICD: implantable cardioverter defibrillator.

**Table 2:** Early outcomes of AF patients undergoing TMVR between 2016 and 2019.

Clinical outcomes	2016	2017	2018	2019	*P*-trend
Number	1,342	1,973	2,422	3,458	0.015
In-hospital outcomes					
Death in hospital	0.026	0.024	0.021	0.018	0.049
Transfusion	0.045	0.062	0.047	0.045	0.193
Sepsis	0.027	0.029	0.021	0.020	0.037
Acute kidney injury	0.184	0.182	0.191	0.196	0.196
Mechanical ventilation	0.016	0.016	0.014	0.011	0.128
Stroke	0.011	0.011	0.011	0.008	0.610
Length of stay	3 [2, 7]	2 [1, 6]	2 [1, 5]	2 [1, 4]	<0.001
30-day readmission rate					
All cause	0.164	0.171	0.178	0.145	0.074
Cardiovascular	0.086	0.076	0.084	0.076	0.464
Heart failure	0.047	0.023	0.019	0.020	<0.001

Nonnormally distributed continuous variables were expressed by medians (interquartile range (IQR)) and categorical variables were expressed as percentages.

**Table 3:** Early outcomes of AF patients undergoing SMVR between 2016 and 2019.

Clinical outcomes	2016	2017	2018	2019	*P*-trend
Number	4323	4462	4103	4084	0.230
In-hospital outcomes					
In-hospital death	0.022	0.020	0.015	0.017	0.037
Transfusion	0.149	0.160	0.153	0.143	0.405
Sepsis	0.031	0.028	0.027	0.033	0.723
Acute kidney injury	0.157	0.158	0.178	0.170	0.181
Mechanical ventilation	0.030	0.033	0.023	0.027	0.089
Stroke	0.024	0.023	0.023	0.023	0.653
Length of stay	7 [5, 12]	7 [5, 11]	7 [5, 10]	7 [5, 11]	0.613
30-day readmission rate					
All-cause	0.143	0.126	0.128	0.120	0.011
Cardiovascular	0.075	0.065	0.069	0.066	<0.001
Heart failure	0.025	0.015	0.013	0.012	<0.001

Nonnormally distributed continuous variables were expressed by medians (interquartile range (IQR)) and categorical variables were expressed as percentages.

**Table 4:** Factors associated with in-hospital death undergoing TMVR.

Variables	Univariate logistic regression			Multivariate logistic regression		
	OR	95% CI	*P* value	OR	95% CI	*P* value
LOS >5 days	8.54	5.55-13.15	<0.001	5.29	3.16–8.87	<0.001
Dyslipidemia	0.46	0.31–0.69	<0.001	0.46	0.3–0.69	<0.001
Cerebrovascular disease	3.16	1.85–5.39	<0.001	2.78	1.46–5.3	0.002
Heart failure with reduced ejection fraction	2.06	1.1–3.89	0.02	2.32	1.22–4.44	0.011
Urgent/emergent admission	4.57	3.09–6.78	<0.001	1.78	1.11–2.86	0.018
Heart failure with preserved ejection fraction	1.82	0.95–3.49	0.07	1.93	0.99–3.75	0.054
Renal disease	1.9	1.3–2.8	<0.001	1.66	0.95–2.87	0.073
Smoke	0.57	0.36–0.91	0.02	0.68	0.42–1.1	0.116
Charlson comorbidity index	1.15	1.05–1.27	<0.001	0.89	0.76–1.04	0.143
Paraplegia	6.18	2.38-16.07	<0.001	1.63	0.51–5.17	0.407
Myocardial infarction	1.7	1.02–2.85	0.040	1.22	0.69–2.15	0.498
Age >75	0.78	0.53–1.14	0.190			
AIDS	NA	NA	NA			
Alcohol use	NA	NA	0.980			
Anemia	1.49	0.68–3.25	0.310			
Chronic pulmonary disease	0.9	0.58–1.42	0.660			
Dementia	0.46	0.06–3.33	0.440			
Diabetes	0.76	0.42–1.37	0.360			
Female	0.91	0.62–1.34	0.640			
Malignant cancer	1.07	0.39–2.94	0.890			
Metastatic solid tumor	0	0-Inf	0.980			
Liver disease	1.11	0.35–3.54	0.860			
Obesity	0.72	0.38–1.35	0.300			
Paroxysmal AF	0.96	0.64–1.43	0.830			
Peptic ulcer disease	1.14	0.41–3.13	0.80			
Peripheral vascular disease	1.3	0.81–2.07	0.280			
Prior CABD	0.84	0.41–1.75	0.650			
Prior PCI	0.57	0.26–1.23	0.150			
Prior PPM/ICD	0.73	0.4–1.31	0.290			
Prior stroke	0.72	0.36–1.43	0.340			
Rheumatic disease	1.44	0.84–2.47	0.190			

OR: odds ratio; CI: confidence interval; LOS: length of stay; AF: atrial fibrillation; CABG: coronary artery bypass graft surgery; SMVR: surgical mitral valve repair; TMVR: transcatheter mitral valve repair; PPM: permanent pacemaker implant; ICD: implantable cardioverter defibrillator.

**Table 5:** Factors associated with 30-day readmission after TMVR.

Variables	Univariate logistic regression			Multivariate logistic regression		
	OR	95% CI	*P* value	OR	95% CI	*P* value
LOS>5 day	2.69	2.27–3.18	<0.001	2.15	1.75–2.65	<0.001
Renal disease	1.48	1.26–1.74	<0.001	1.37	1.14–1.64	<0.001
Heart failure with reduced ejection fraction	1.92	1.33–2.77	<0.001	1.87	1.25–2.78	0.0022
Chronic pulmonary disease	1.5	1.26–1.78	<0.001	1.34	1.1–1.62	0.003
Female	1.26	1.07–1.48	<0.001	1.23	1.04–1.46	0.014
Dementia	1.95	1.21–3.12	0.01	1.68	1.03–2.76	0.040
Elective	1.99	1.68–2.36	<0.001	1.22	0.99–1.5	0.059
Heart failure with preserved ejection fraction	1.9	1.09–3.32	0.02	1.39	0.78–2.47	0.267
Charlson comorbidity index	1.13	1.09–1.18	<0.001	1.02	0.95–1.09	0.640
Age>75	0.91	0.77–1.07	0.24			
AIDS	2.72	0.5–14.85	0.25			
Alcohol use	1.08	0.13–9.3	0.94			
Anemia	1.29	0.9–1.85	0.16			
Cerebrovascular disease	1.12	0.8–1.57	0.5			
Diabetes	1.02	0.82–1.27	0.86			
Dyslipidemia	0.87	0.74–1.02	0.09			
Metastatic solid tumor	1.94	0.7–5.41	0.2			
Liver disease	1.06	0.65–1.75	0.81			
Myocardial infarction	1.06	0.82–1.37	0.66			
Obesity	1.01	0.8–1.28	0.91			
Paraplegia	1.56	0.71–3.43	0.27			
Paroxysmal AF	1.05	0.89–1.23	0.59			
Peptic ulcer disease	1.48	0.99–2.2	0.05			
Peripheral vascular disease	1.07	0.87–1.31	0.54			
Prior CABG	0.87	0.65–1.17	0.37			
Prior PCI	0.78	0.59–1.02	0.07			
Prior PPM/ICD	1.03	0.83–1.28	0.81			
Prior stroke	1.16	0.91–1.48	0.24			
Rheumatic disease	0.74	0.22–2.47	0.62			
Smoke	0.87	0.73–1.03	0.11			

OR: odds ratio; CI: confidence interval; LOS: length of stay; AF, atrial fibrillation; CABG: coronary artery bypass graft surgery; SMVR, surgical mitral valve repair; TMVR: transcatheter mitral valve repair; PPM: permanent pacemaker implant; ICD: implantable cardioverter defibrillator.

**Table 6:** In-hospital and 30-day outcomes for SMVR versus TMVR in patients with AF in unmatched and matched cohorts.

	Unmatched cohort		*P* value	Matched cohort		*P* value
	SMVR in AF	TMVR in AF		SMVR in AF	TMVR in AF	
Number	16,972	9,195		4,680	4,680	
In-hospital death	1.9%	2.1%	0.166	3.3%	2.3%	0.004
Transfusion	15.1%	4.9%	<0.001	20.5%	4.6%	<0.001
Acute kidney injury	18.8%	16.7%	<0.001	29.2%	15.2%	<0.001
Sepsis	3.0%	2.3%	0.002	3.8%	2.8%	0.008
Cardiogenic shock	9.1%	5.2%	<0.001	11.7%	6.0%	<0.001
Mechanical ventilation	2.8%	1.4%	<0.001	4.0%	1.8%	<0.001
Outcome stroke	2.3%	1.0%	<0.001	2.0%	1.5%	0.058
Length of stay	7.00 [5.00, 11.00]	2.00 [1.00, 5.00]	<0.001	8.00 [6.00, 14.00]	2.00 [1.00, 6.00]	<0.001
Cost	44408 [29620, 70491]	42349 [28822, 65325]	<0.001	49831 [32735, 80787]	42336 [28219, 64985]	<0.001
30-day readmission						
All-cause	12.8%	15.8%	<0.001	16.2%	15.2%	0.286
Cardiovascular	6.7%	7.6%	<0.001	8.2%	7.2%	0.094
Heart failure	1.6%	2.4%	<0.001	2.9%	2.7%	0.466

Nonnormally distributed continuous variables were expressed by medians (interquartile range (IQR)) and categorical variables were expressed as percentages. AF: atrial fibrillation; SMVR: surgical mitral valve repair; TMVR: transcatheter mitral valve repair.

**Table 7:** In-hospital outcomes in SMVR versus TMVR in atrial fibrillation in the propensity score matched cohort.

Variables	OR (95% CI)	*P* value
In-hospital death	0.69 (0.53, 0.88)	0.003
Transfusion	0.19 (0.16, 0.22)	<0.001
Acute kidney injury	0.44 (0.39, 0.48)	<0.001
Sepsis	0.73 (0.58, 0.91)	0.007
Cardiogenic shock	0.49 (0.42, 0.56)	<0.001
Mechanical ventilation	0.45 (0.34, 0.58)	<0.001
Stroke	0.73 (0.53, 1.00)	0.049

OR: odds ratio; CI: confidence interval; AF: atrial fibrillation; SMVR: surgical mitral valve repair; TMVR: transcatheter mitral valve repair.

**Table 8:** In-hospital outcomes in SMVR versus TMVR in paroxysmal AF in the propensity score matched cohort.

	SMVR	TMVR	OR (95% CI)	*P* value
Number	1,938	1,751		
In-hospital death	3.1%	2.2%	0.70 (0.46, 1.05)	0.087
Transfusion	21.6%	4.6%	0.18 (0.14, 0.22)	<0.001
Acute kidney injury	29.9%	16.9%	0.48 (0.41, 0.56)	<0.001
Sepsis	4.4%	2.9%	0.64 (0.45, 0.91)	0.014
Cardiogenic shock	11.8%	6.4%	0.51 (0.40, 0.64)	<0.001
Mechanical ventilation	3.9%	2.1%	0.52 (0.34, 0.77)	0.002
Stroke	2.4%	1.9%	0.80 (0.51, 1.24)	0.318

OR: odds ratio; CI: confidence interval; AF: atrial fibrillation; SMVR: surgical mitral valve repair; TMVR: transcatheter mitral valve repair.

**Table 9:** In-hospital outcomes in SMVR versus TMVR in nonparoxysmal AF in the propensity score matched cohort.

	SMVR	TMVR	OR (95% CI)	*P* value
Number	2,742	2,929		
In-hospital death	3.4%	2.3%	0.68 (0.49, 0.93)	
Transfusion	19.7%	4.6%	0.18 (0.14, 0.22)	<0.001
Acute kidney injury	28.7%	14.2%	0.48 (0.41, 0.56)	<0.001
Sepsis	3.4%	2.7%	0.64 (0.45, 0.91)	0.014
Cardiogenic shock	11.6%	5.8%	0.51 (0.40, 0.64)	<0.001
Mechanical ventilation	4.0%	1.7%	0.52 (0.34, 0.77)	0.002
Stroke	1.7%	1.2%	0.80 (0.51, 1.24)	0.318

OR: odds ratio; CI: confidence interval; AF: atrial fibrillation; SMVR: surgical mitral valve repair; TMVR: transcatheter mitral valve repair.

**Table 10:** In-hospital outcomes in SMVR versus TMVR in the propensity score matched cohort with different etiologies of mitral regurgitation.

	SMVR	TMVR	OR (95% CI)	*P* value
Rheumatic mitral				
Number	341	511		
In-hospital death	6.2%	4.3%	0.69 (0.37, 1.27)	0.228
Transfusion	24.6%	8.6%	0.29 (0.19, 0.43)	<0.001
Acute kidney injury	34.9%	28.4%	0.74 (0.55, 0.99)	0.044
Sepsis	26.5%	5.7%	0.87 (0.49, 1.56)	0.640
Cardiogenic shock	16.1%	12.9%	0.77 (0.52, 1.14)	0.189
Mechanical ventilation	7.3%	3.9%	0.51 (0.28, 0.94)	0.031
Stroke	1.8%	2.3%	1.34 (0.52, 3.89)	0.559
Mitral prolapse				
Number	116	448		
In-hospital death	1.7%	1.8%	1.04 (0.26, 6.93)	0.964
Transfusion	12.9%	2.9%	0.20 (0.09, 0.44)	<0.001
Acute kidney injury	17.2%	10.9%	0.59 (0.34, 1.06)	0.067
Sepsis	3.4%	1.3%	0.38 (0.11, 1.51)	0.139
Cardiogenic shock	11.2%	4.9%	0.41 (0.20, 0.86)	0.015
Mechanical ventilation	3.4%	1.6%	0.44 (0.13, 1.72)	0.202
Stroke	0.9%	1.8%	2.09 (0.38, 38.99)	0.489
Congenital valvular heart disease				
Number	16	11		
In-hospital death	6.2%	0	NA	NA
Transfusion	37.5%	9.1%	0.17 (0.01, 1.23)	0.125
Acute kidney injury	25.0%	27.3%	1.12 (0.18, 6.52)	0.895
Sepsis	6.2%	0	NA	NA
Cardiogenic shock	18.8%	18.2%	0.96 (0.11, 7.00)	0.970
Mechanical ventilation	6.2%	9.1%	1.50 (0.05, 40.98)	0.783
Stroke	6.2%	0%	NA	NA
Other reason				
Number	4,212	3,730		
In-hospital death	3.1%	2.1%	0.66 (0.50, 0.88)	0.005
Transfusion	20.3%	4.3%	0.18 (0.15, 0.21)	<0.001
Acute kidney injury	29.1%	14.0%	0.40 (0.35, 0.44)	<0.001
Sepsis	3.6%	2.5%	NA	NA
Cardiogenic shock	11.3%	5.3%	0.43 (0.36, 0.52)	<0.001
Mechanical ventilation	3.7%	1.6%	0.41 (0.30, 0.55)	<0.001
Stroke	2.0%	1.3%	0.64 (0.45, 0.91)	<0.001

OR: odds ratio; CI: confidence interval; SMVR: surgical mitral valve repair; TMVR: transcatheter mitral valve repair.

## Data Availability

The data used to support the findings of this study are available on request from the corresponding author.

## Supplementary Materials

Supplementary Materials Supplement Table 1: list of ICD-10 used in this article. Supplement Table 2: variables used in propensity-match analysis. Supplementary Figure 1: data distribution before and after propensity matching.
