# Supplementary material for: Temporal Trends and Early Outcomes of Transcatheter versus Surgical Mitral Valve Repair in Atrial Fibrillation Patients
Source: J Interv Cardiol. 2023 Oct 12;2023:4332684. doi: 10.1155/2023/4332684 (PMC10586899; doi:10.1155/2023/4332684)
Supplement: Supplementary Materials — Supplement Table 1: list of ICD-10 used in this article. Supplement Table 2: variables used in propensity-match analysis. Supplementary Figure 1: data distribution before and after propensity matching. [file 4332684.f1.rtf]

Supplementary Material

Supplementary table 1: List of the used ICD-10 codes AF = atrial fibrillation; AVR = mitral valve repair; CABG = coronary artery bypass grafting; SMVR = surgical mitral valve repair; TMVR = transcatheter mitral valve repair; PPM = permanent pacemaker; ICD = implantable cardioverter-defibrillator£»SMD = Standard Mean Difference
	ICD 10 CODE	
TMVR		02UG3JZ, 02UG4KZ	
SMVR		02QG0ZE, 02QG0ZZ 	
AF		I48, I480, I481, I4811, I4819, 
I482, I4820, I4821, I4891	
CABG		See below
	
Infective endocarditis		B376,A3282,A3951,A5203,B3321,I330,I339,I38 ,I39	
Prior CABG		Z951	
Prior PCI		Z955,Z9861	
Prior PPM/ICD		Z950,Z95810	
Hemopericardium		I312	
Respiratory complications		J9562,J9561,J9572,J9571,J9588,J95861,J95860,J95831,J95830,J95863,J95862,J9589,J95821,J95822	
Blood transfusion	30243N0, 30243N1, 30243P0, 30243P1, 30243H0, 30243H1, 30240N0, 30240N1, 30240P0, 30240P1, 30240H0, 30240H1, 30230H0, 30230H1, 30230N0, 30230N1, 30230P0, 30230P1, 30233N0, 30233N1, 30233P0, 
30233P1	
Acute kidney injury	N170, N171, N172, N178, N179, N19, N990, R34, R944	
Cardiac complication	T8201XA, T8202XA, T8203XA,
T8209XA, T82222A, T82223A,
T82228A, T82867A, T82897A,
T82897A, I97710, I97790, I9788,
I9789, I9781, I9782	
Cardiac tamponade 	I314	
Cardiac arrest	I46, I469, I462, I9712, I9771, I97710, I97711, I97121	
Cardiogenic shock	R570,T8111XA	
Permanent pacemaker	02HK3JZ, 02H63JZ, 02HN0JZ, 02H60JZ, 02H60NZ, 02H63JZ, 02H63NZ, 02H64JZ, 02H64NZ, 02HK0JZ, 02HK0NZ, 02HK3JZ, 02HK3NZ, 02HK4JZ, 02HK4NZ,  02HN4JZ, 0JH604Z, 0JH634Z, 0JH605Z, 0JH607Z, 0JH635Z, 0JH606Z, 0JH634Z, 0JH635Z, 0JH636Z, 0JH637Z	
Acquired pneumonia	J13,J14,J150,J151,J1520,J15211,J15212,J1529,J153,J154,J155,J156,J157,J158,J159,J160,J168,J17,J180,J181,J182,J188,J189	
Sepsis	T8140XA,T8140XD,T8140XS,T8141XA,T8141XD,T8141XS,T8142XA,T8142XD,T8142XS,T8143XA,T8143XD,T8143XS,T8144XA,T8144XD,T8144XS,T8149XA,T8149XD,T8149XS,T827XXA,T827XXD,T827XXS,T83510A,T83510D,T83510S,T83511A,T83511D,T83511S,T83512A,T83512D,T83512S,T83518A,T83518D,T83518S,T83590A,T83590D,T83590S,T83591A,T83591D,T83591S,T83592A,T83592D,T83592S,T83593A,T83593D,T83593S,T83598A,T83598D,T83598S,T8571XA,T8571XD,T8571XS,T8572XA,T8572XD,T8572XS,T85730A,T85730D,T85730S,T85731A,T85731D,T85731S,T85732A,T85732D,T85732S,T85733A,T85733D,T85733S,T85734A,T85734D,T85734S,T85735A,T85735D,T85735S,T85738A,T85738D,T85738S,T8579XA,T8579XD,T8579XS,A4101,A4102,A411,A412,A413,A414,A4150,A4151,A4152,A4153,A4159,A4181,A4189,A419,A400,A401,R6520,T8351XA,T80219A,T80211A,A403,A408,A409,B377	
Mechanical ventilation	5A1955Z	
Tracheostomy	0B110F4, 0B110Z4, 0B113F4, 0B113Z4
0B114F4, 0B114Z4	
Vascular complications 	S25401A,S25401D,S25401S,S25402A,S25402D,S25402S,S25409A,S25409D,S25409S,S25411A,S25411D,S25411S,S25412A,S25412D,S25412S,S25419A,S25419D,S25419S,S25421A,S25421D,S25421S,S25422A,S25422D,S25422S,S25429A,S25429D,S25429S,S25491A,S25491D,S25491S,S25492A,S25492D,S25492S,S25499A,S25499D,S25499S,S3510XA,S3510XD,S3510XS,S3511XA,S3511XD,S3511XS,S3512XA,S3512XD,S3512XS,S3519XA,S3519XD,S3519XS,S35515A,S35515D,S35515S,S35516A,S35516D,S35516S,S75101A,S75101D,S75101S,S75102A,S75102D,S75102S,S75109A,S75109D,S75109S,S75111A,S75111D,S75111S,S75112A,S75112D,S75112S,S75119A,S75119D,S75119S,S75121A,S75121D,S75121S,S75122A,S75122D,S75122S,S75129A,S75129D,S75129S,S75191A,S75191D,S75191S,S75192A,S75192D,S75192S,S75199A,S75199D,S75199S	
Pleural Effusion	J90,J918	
Pericardiocentesis	0W9D30Z,0W9D3ZX,0W9D3ZZ,0W9D40Z,0W9D4ZX,0W9D4ZZ	


CABG: 0021, 0210083, 0210088, 0210089, 021008C, 021008F, 021008W, 0210093, 0210098, 0210099, 021009C, 021009F, 021009W, 02100A3, 02100A8, 02100A9, 02100AC, 02100AF, 02100AW, 02100J3, 02100J8, 02100J9, 02100JC, 02100JF, 02100JW, 02100K3, 02100K8, 02100K9, 02100KC, 02100KF, 02100KW, 02100Z3, 02100Z8, 02100Z9, 02100ZC, 02100ZF, 0210344, 02103D4, 0210444, 0210483, 0210488, 0210489, 021048C, 021048F, 021048W, 0210493, 0210498, 0210499, 021049C, 021049F, 021049W, 02104A3, 02110Z9, 02104A8, 02104A9, 02104AC, 02104AF, 02104AW, 02104D4, 02104J3, 02104J8, 02104J9, 02104JC, 02104JF, 02104JW, 02104K3, 02104K8, 02104K9, 02104KC, 02104KF, 02104KW, 02104Z3, 02104Z8, 02104Z9, 02104ZC, 02104ZF, 0211, 0211083, 0211088, 0211089, 021108C, 021108F, 021108W, 0211093, 0211098, 0211099, 021109C, 021109F, 021109W, 02110A3, 02110A8, 02110A9, 02110AC, 02110AF, 02110AW, 02110J3, 02110J8, 02110J9, 02110JC, 02110JF, 02110JW, 02110K3, 02110K8, 02110K9, 02110KC, 02110KF, 02110KW, 02110Z3, 02110Z8, 02110ZC, 02110ZF, 0211344, 02113D4, 0211444, 0211483, 0211488, 0211489, 021148C, 021148F, 021148W, 0211493, 0211498, 0211499, 021149C, 021149F, 021149W, 02114A3, 02114A8, 02114A9, 02114AC, 02114AF, 02114AW, 02114D4, 02114J3, 02114J8, 02114J9, 02114JC, 02114JF, 02114JW, 02114K3, 02114K8, 02114K9, 02114KC, 02114KF, 02114KW, 02114Z3, 02114Z8, 02114Z9, 02114ZC, 02114ZF, 0212, 0212083, 0212088, 0212089, 021208C, 021208F, 021208W, 0212093, 0212098, 0212099, 021209C, 021209F, 021209W, 02120A3, 02120A8, 02120A9, 02120AC, 02120AF, 02120AW, 02120J3, 02120J8, 02120J9, 02120JC, 02120JF, 02120JW, 02120K3, 02120K8, 02120K9, 02120KC, 02120KF, 02120KW, 02120Z3, 02120Z8, 02120Z9, 02120ZC, 02120ZF, 0212344, 02123D4, 0212444, 0212483, 0212488, 0212489, 021248C, 021248F, 021248W, 0212493, 0212498, 0212499, 021249C, 021249F, 021249W, 02124A3, 02124A8, 02124A9, 02124AC, 02124AF, 02124AW, 02124D4, 02124J3, 02124J8, 02124J9, 02124JC, 02124JF, 02124JW, 02124K3, 02124K8, 02124K9, 02124KC, 02124KF, 02124KW, 02124Z3, 02124Z8, 02124Z9, 02124ZC, 02124ZF, 0213, 0213083, 0213088, 0213089, 021308C, 021308F, 021308W, 0213093, 0213098, 0213099, 021309C, 021309F, 021309W, 02130A3, 02130A8, 02130A9, 02130AC, 02130AF, 02130AW, 02130J3, 02130J8, 02130J9, 02130JC, 02130JF, 02130JW, 02130K3, 02130K8, 02130K9, 02130KC, 02130KF, 02130KW, 02130Z3, 02130Z8, 02130Z9, 02130ZC, 02130ZF, 0213344, 02133D4, 0213444, 0213483, 0213488, 0213489, 021348C, 021348F, 021348W, 0213493, 0213498, 0213499, 021349C, 021349F, 021349W, 02134A3, 02134A8, 02134A9, 02134AC, 02134AF, 02134AW, 02134D4, 02134J3, 02134J8, 02134J9, 02134JC, 02134JF, 02134JW, 02134K3, 02134K8, 02134K9, 02134KC, 02134KF, 02134KW, 02134Z3, 02134Z8, 02134Z9, 02134ZC, 02134ZF


Supplementary table 2: Variables used in Propensity match analysis 
1. Age
2. Female
3. Prior_PCI
4. Prior_CABG
5. Prior_PPM_ICD
6. Smoke
7. Dyslipidemia
8. Anemia
9. Obesity
10. Alcohol_use
11. Elective
12. Stroke
13. Income
14. Pay
15. Race
16. Hosp_bedsize
17. Hosp_locteach
18. Myocardial_infarct
19. Congestive_heart_failure
20. Peripheral_vascular_disease
21.Cerebrovascular_disease
22. Dementia
23. Chronic_pulmonary_disease
24. Rheumatic_disease
25. Peptic_ulcer_disease
26. Mild_liver_disease
27.Diabetes,Paraplegia
28. Renal_disease
29. Malignant_cancer
30. Severe_liver_disease
31. Metastatic_solid_tumor


Supplementary Figure 1: Data distribution before and after propensity matching
